# Bone Pain Is Associated with Short Radiographic Progression-Free Survival in Patients with Metastatic Castration-Sensitive Prostate Cancer

**DOI:** 10.3390/jcm15103968

**Published:** 2026-05-21

**Authors:** Aykut Özmen, Caner Kapar, İlkay Gültürk, Selçuk Şahin, Kamil Gökhan Şeker, Volkan Tuğcu, Deniz Tural

**Affiliations:** 1 Department of Medical Oncology, Başakşehir Çam and Sakura City Hospital, Istanbul 34480, Turkey; 2 Department of Medical Oncology, Bakirköy Research and Education Hospital, Istanbul 34140, Turkey; kaparcaner@gmail.com; 3 Department of Medical Oncology, Istanbul Research and Education Hospital, Istanbul 34098, Turkey; gulturkilkay@gmail.com; 4 Urology Department, Istinye University, Istanbul 34010, Turkey; urosahin@gmail.com (S.Ş.); gkhnseker@hotmail.com (K.G.Ş.); volkan.tugcu@istinye.edu.tr (V.T.); 5 Department of Medical Oncology, Koç University Hospital, Istanbul 34010, Turkey; deniztural@gmail.com

**Keywords:** prostate cancer, bone pain, prognostic factors, radiographic progression-free survival

## Abstract

**Background:** We aimed to evaluate the prognostic significance of bone pain at presentation in metastatic castration-sensitive prostate cancer (mCSPC) and its association with radiographic progression-free survival (rPFS), independent of established prognostic factors. **Methods:** We retrospectively analyzed 205 patients with mCSPC treated at our center. Data were obtained from patient files and hospital records. rPFS was estimated using the Kaplan–Meier method and compared using the log-rank test. Baseline characteristics according to bone pain status were compared using the χ^2^ test for categorical variables and the Wilcoxon test for continuous variables. **Results:** Median rPFS for the entire cohort was 23.8 months (95% CI: 18.6–29.0). In univariate analysis, median rPFS was significantly shorter in patients with bone pain compared to those without (16.9 vs. 29.5 months, *p* < 0.001). Other factors associated with worse rPFS included ECOG PS ≥ 1, DNA repair mutations, high disease volume, liver metastasis, hemoglobin < 12 g/dL, and albumin < 3.5 g/dL. In multivariate analysis, bone pain (HR = 2.37, 95% CI: 1.29–4.34; *p* = 0.005), albumin < 3.5 g/dL (HR = 1.99; *p* = 0.034), hemoglobin < 12 g/dL (HR = 2.00; *p* = 0.005), and liver metastasis (HR = 3.07; *p* = 0.024) remained independent predictors of shorter rPFS. **Conclusions:** Bone pain at presentation is an independent prognostic factor for shorter rPFS and may help guide risk stratification and treatment decisions in mCSPC.

## 1. Introduction

Baseline biomarkers have been shown to predict survival outcomes and may allow for more personalized treatment strategies in metastatic prostate cancer [1]. Both prognostic and predictive factors for survival have been well-established in this setting [2,3]. Previous studies have identified several independent prognostic variables in metastatic castration-resistant prostate cancer (mCRPC), including bone pain, Eastern Cooperative Oncology Group (ECOG) performance status (PS), Gleason score, hemoglobin (Hb) level, prostate-specific antigen (PSA) level, alkaline phosphatase (ALP), lactate dehydrogenase (LDH), and the presence of visceral disease [3,4].

Bone pain is one of the most common and clinically relevant symptoms in metastatic prostate cancer and is generally associated with skeletal metastatic involvement, impaired quality of life, and increased disease burden. In routine clinical practice, cancer-related bone pain is commonly assessed using patient-reported symptoms and analgesic requirements, while standardized instruments such as the Brief Pain Inventory (BPI) may also be used in prospective studies.

Several investigations have also confirmed that pain is a statistically significant predictor of overall survival (OS) in men with mCRPC [1,2]. Bone metastases represent the most frequent metastatic site in castration-sensitive prostate cancer, observed in nearly 80% of patients at diagnosis [5]. Despite this, evidence regarding the impact of bone pain on survival outcomes in patients with metastatic castration-sensitive prostate cancer (mCSPC) remains limited [5,6].

Given this gap, we aimed to assess the prognostic value of bone pain at the time of diagnosis in mCSPC. Establishing that bone pain carries prognostic significance independent of other recognized factors could help inform the selection of more intensive approaches in future trials, such as triplet therapies incorporating docetaxel and androgen receptor pathway inhibitors (ARPIs). Moreover, integrating bone pain at presentation into clinical studies and risk-stratification models may improve the accuracy of prognostic scoring systems. In this study, we specifically examined the association between bone pain and radiographic progression-free survival (rPFS) in patients with mCSPC.

## 2. Materials and Methods

### 2.1. Study Design

We evaluated retrospective data from 205 patients between 15 June 2017 and 15 March 2024 with mCSPC treated at Bakırköy Research and Education Hospital Oncology Center. Patient data were obtained from patient files and hospital records. Demographic, clinicopathological, laboratory, and clinical outcome data were collected in a single central database retrospectively.

### 2.2. Data Collection

The following eleven potential prognostic factors were included in the univariate analyses: Presentation with pain at the time of metastatic disease, age (<70 vs. ≥70 years), ECOG PS (0 vs. ≥1), Gleason score (<8 vs. ≥8), Hb (normal vs. abnormal), PSA level at the time of diagnosis, ALP (normal vs. abnormal), LDH (normal vs. abnormal), bone metastases (yes vs. no), visceral metastases (yes vs. no), metastases at diagnosis vs. after local treatment failure, and high vs. low volume based on the CHAARTED criteria [7].

Bone pain status was determined retrospectively based on documented patient-reported pain symptoms attributed to sites of radiographically confirmed bone metastases at the time of mCSPC diagnosis. Patients requiring analgesics or opioid medications for cancer-related bone pain were classified into the pain-presenting group. Analgesic use was assessed at baseline, defined as the time of mCSPC diagnosis before initiation of systemic treatment. Standardized pain assessment scales were not routinely used because of the retrospective nature of the study.

LDH, ALP, and Hb were defined as abnormal for values above the upper limit or below the lower limit of the normal range, as determined by the laboratory performing the assays. Albumin, LDH, ALP and Hb were classified as abnormal based on predefined thresholds. Albumin levels below 3.5 g/dL (normal range: 3.5–5.0 g/dL) were considered low. LDH values greater than 1.5 times the upper limit of normal (ULN; reference range: 140–280 U/L) were defined as high, and ALP values above the ULN (reference range: 44–147 U/L) were also considered high. Hb, LDH, and ALP were otherwise considered abnormal when outside the normal ranges established by the performing laboratory. PSA level was evaluated according to the median value.

### 2.3. Statistical Analysis

The primary endpoint was radiographic progression-free survival (rPFS). It was defined as the time interval from treatment initiation to the date when the first site of disease progression or death occurs, whichever comes first, according to the Prostate Cancer Clinical Trials Working Group 3 criteria [8]. rPFS was estimated using the Kaplan–Meier method, and the log-rank test was used for comparisons between different subgroups. Differences in baseline characteristics by bone pain status at mCSPC diagnosis were evaluated using the χ^2^ test for categorical variables and the Wilcoxon test for continuous variables. Univariate analysis was performed to identify clinical and laboratory factors associated with rPFS. Statistical significance was defined as *p* < 0.05. Variables that were statistically significant in univariate analysis, as well as clinically relevant variables with borderline statistical significance, were included in the multivariate model. Variables included in the multivariate model were bone pain, disease volume, liver metastasis, hemoglobin level, albumin level, and ALP. Missing data were handled using available-case analysis, and no imputation method was applied because of the retrospective nature of the study. All data analyses were performed using SPSS version 23 (IBM Corp., Armonk, NY, USA).

## 3. Results

### 3.1. Patient Characteristics

Data from 205 patients were analyzed. Median follow-up time was 32.3 months (range, 3–86 months) and median age was 68 years (range, 38–91 years). A detailed summary of the patient characteristics is shown in Table 1. Patients with bone pain presented significantly more frequently with ECOG PS ≥ 1, metastases at diagnosis, high disease volume and liver metastases. Other differences with a trend toward statistical significance included more metachronous metastases, lower Hb levels, higher PSA levels and higher docetaxel use in patients presenting with bone pain. Additionally, DNA repair mutations were evaluated in 91 of the 205 patients. The mutation rate was 9.6% in patients presenting with pain and 5% those without pain; however, this numerical difference was not statistically significant.

### 3.2. Analysis of rPFS and Prognostic Factors

**Figure 1:**
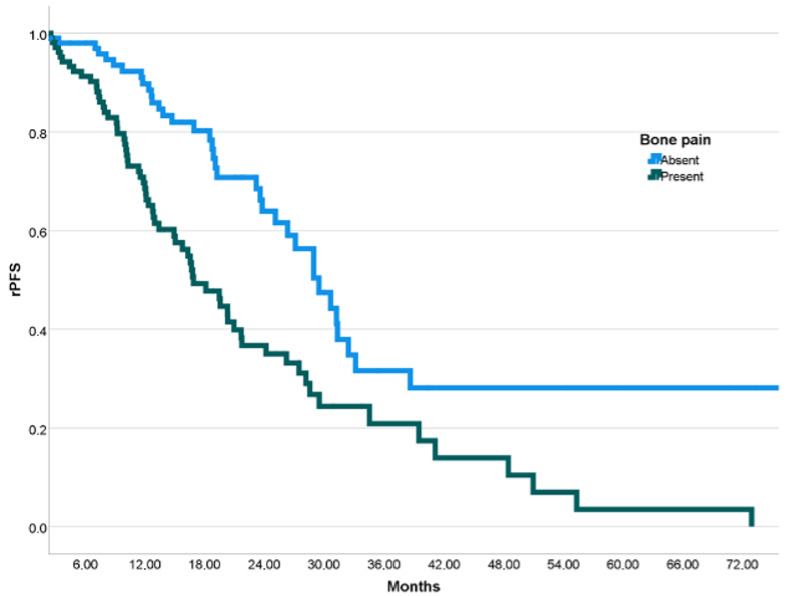
Kaplan–Meier curves of radiographic progression-free survival (rPFS) stratified by bone pain.

Median rPFS of all patients was 23.8 months (95% CI: 18.6–29.0 months). In univariate analysis, median rPFS was 16.9 months vs. 29.5 months in patients with bone pain vs. those without (*p* = 0.001) (Figure 1, Table 2). Median rPFS was 29.0 vs. 20.3 months in patients with ECOG PS 0 vs. ≥1 (*p* = 0.094), 21.7 vs. 13.8 months in patients with absent vs. present DNA repair mutations (*p* = 0.064), 27.1 vs. 19.5 months in patients with low vs. high disease volume (*p* = 0.050), 25.1 vs. 14.9 months in patients without vs. with liver metastases (*p* = 0.011), 27.5 vs. 16.9 months in patients with Hb ≥ 12 vs. <12 g/dL (*p* = 0.002), and 27.1 vs. 14.7 months in patients with albumin ≥3.5 vs. <3.5 g/dL (*p* = 0.002), respectively (Table 2).

**Figure 2:**
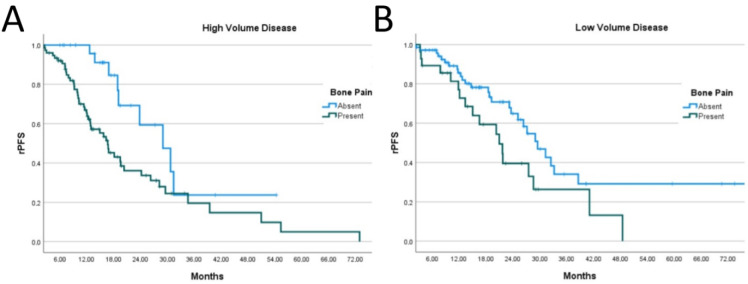
Kaplan–Meier curves of radiographic progression-free survival (rPFS) according to bone pain status in patients with (**A**) high-volume disease and (**B**) low-volume disease.

In the high-volume subgroup, median rPFS was 16.7 months in patients presenting with pain compared to 29.0 months in those with no pain, which was statistically significant (*p* < 0.001) (Figure 2A, Table 2). Similarly, rPFS was 21.0 months in patients with low volume and pain compared to 29.50 months in those with low volume and no pain, with a statistically significant difference (*p* < 0.001) (Figure 2B, Table 2).

In multivariate analysis, bone pain (HR = 2.37, 95% CI: 1.29–4.34; *p* = 0.005), Hb < 12 g/dL (HR = 2.00, 95% CI: 1.90–3.10; *p* = 0.005), albumin < 3.5 g/dL (HR = 1.99, 95% CI: 1.06–3.76; *p* = 0.034) and liver metastasis (HR = 3.07, 95% CI: 1.16–8.15; *p* = 0.024) were identified as independent determinants of shorter rPFS (Table 3).

## 4. Discussion

Approximately 10% of prostate cancer patients present with metastatic disease at the time of initial diagnosis, and their estimated five-year survival rate is around 37% [9]. For most men with mCSPC, international guidelines recommend androgen deprivation therapy (ADT) in combination with an androgen receptor pathway inhibitor (ARPI), with or without the addition of docetaxel chemotherapy [10]. In a pivotal trial of 1199 patients with newly diagnosed metastatic disease, the addition of abiraterone to ADT significantly improved overall survival compared with ADT alone (53.3 months vs. 36.5 months; HR = 0.66, 95% CI: 0.56–0.78) [11]. Similar benefits have been observed with other ARPIs, including enzalutamide and apalutamide, when combined with ADT. Consequently, abiraterone, enzalutamide, and apalutamide are all considered standard treatment options for men with newly diagnosed mCSPC [11,12,13,14,15].

In patients with mCSPC, combining ADT with both ARPIs and docetaxel has demonstrated greater efficacy than ADT plus docetaxel alone. Based on these findings, triplet therapy with ADT, darolutamide, and docetaxel has received regulatory approval for clinical use [16,17,18].

The association between bone pain and clinical outcomes is well-established in metastatic castration-resistant prostate cancer (mCRPC), where pain at presentation is linked to more aggressive disease biology and inferior responses to chemotherapy and ARPI treatment [3,4,5,6]. In contrast, the prognostic relevance of bone pain in men with treatment-naïve mCSPC remains insufficiently defined and has not been comprehensively studied. In the present study, we found that mCSPC patients who reported bone pain at diagnosis experienced poorer treatment responses and overall outcomes compared with those without pain. Importantly, our analysis demonstrated that pain was an independent prognostic factor associated with reduced survival.

In the SWOG-1216 trial, patients who reported baseline bone pain were generally younger and more likely to present with high-volume disease compared to those without pain. After adjustment for potential confounders, baseline bone pain was independently associated with shorter progression-free survival (PFS) and overall survival (OS) [6]. Our results are consistent with these findings, confirming that bone pain correlates with higher disease burden and shorter rPFS. Building on the SWOG-1216 observations, we further demonstrated that DNA repair gene alterations may be more common in patients presenting with bone pain. This could help explain the more aggressive clinical course observed in this subgroup. Unlike mCRPC, where numerous prognostic and predictive markers have been established, relatively few studies have examined such factors in mCSPC. The GETUG-15 trial identified visceral metastases, bone metastases, ECOG PS (0 vs. 1–2), Hb, ALP, LDH, PSA (≤65 ng/mL vs. >65 ng/mL), and pain intensity as significant predictors of OS [5]. Our findings are consistent with GETUG-15 and additionally confirm bone pain as an independent predictor of shorter rPFS in multivariate analysis.

Differences between our findings and those of previous studies may partly be explained by variations in study populations, treatment strategies, and definitions of pain. For example, the SWOG-1216 trial was a prospective clinical trial with a more standardized patient population and predefined study procedures, whereas our study reflects a real-world retrospective cohort including patients treated with heterogeneous systemic approaches. In addition, prior studies have used different methods for pain assessment, including validated quantitative pain scales, while pain classification in our study was based on clinician-documented symptoms and analgesic requirements. Variations in follow-up duration and disease burden across studies may also have contributed to differences in reported outcomes. Nevertheless, despite these methodological differences, our findings remain consistent with prior evidence, suggesting that bone pain is associated with more aggressive disease biology and poorer clinical outcomes in metastatic prostate cancer.

To further investigate the relationship between pain and disease burden, we stratified patients into high- and low-volume subgroups according to the CHAARTED criteria. In contrast to the SWOG-1216 [6] and GETUG-15 [5] studies, we observed that pain at presentation was associated with worse outcomes in both high- and low-volume groups compared with those without pain. This suggests that pain may serve as a prognostic marker of tumor biology, independent of disease volume. Oudard et al. previously showed that men with mCRPC who were asymptomatic had consistently better OS than symptomatic patients treated with docetaxel [2]. Using an 11-point numerical rating scale, they demonstrated a significant association between pain intensity and OS, with moderate or severe pain conferring a substantially higher risk of death compared to minimal or no pain [2].

The principal limitation of our study is the absence of a standardized pain assessment instrument, such as the Wisconsin Brief Pain Inventory (BPI) [19] or the EORTC QLQ-C30 questionnaire [20], which provide structured tools to categorize and quantify pain. Furthermore, patients were classified according to the presence or absence of pain without distinguishing between those requiring non-opioid analgesics and those requiring opioid medications. In addition, DNA repair mutation data were unavailable for a substantial proportion of patients, which may have limited the reliability of exploratory analyses involving this variable. Different systemic treatment regimens may also have influenced both pain control and rPFS outcomes; however, the sample size was insufficient to perform robust treatment-specific interaction analyses. Future prospective studies incorporating validated quantitative pain assessment tools may help evaluate potential dose–response relationships between pain severity and survival outcomes.

## 5. Conclusions

Our study demonstrates that bone pain at presentation is associated with poor prognostic factors in mCSPC, including high disease volume, poor performance status, and liver metastases. Furthermore, bone pain at presentation was identified as an independent prognostic factor for shorter rPFS in these patients. We also showed that pain is an important prognostic marker independent of disease volume, suggesting that patients with pain may have more aggressive tumor biology. Pain, as an easily measurable parameter, provides important information regarding the course of the disease. Therefore, bone pain at presentation should be considered a prognostic factor when selecting treatment options or developing prognostic models for mCSPC and may help identify patients who could benefit from treatment intensification strategies and closer radiographic surveillance.

## Figures and Tables

**Table 1:** Baseline characteristics of patients.

Variable	Patients		*p*-Value
	Presence of Bone Pain (*n* = 104)	Absence of Bone Pain (*n* = 101)	
**ECOG PS**, ***n* (%)**			<0.001
0	34 (32.7)	67 (66.3)	
≥1	70 (67.3)	34 (33.7)	
**Gleason score at diagnosis**, ***n* (%)**			0.723
<8	31 (29.8)	34 (33.7)	
≥8	66 (63.5)	65 (64.4)	
Unknown	7 (6.7)	2 (1.9)	
**Age**, ***n* (%)**			0.242
<70 years	40 (38.5)	47 (46.5)	
≥70 years	64 (61.5)	54 (53.5)	
**Disease volume**, ***n* (%)**			<0.001
High	76 (73.1)	29 (28.7)	
Low	28 (26.9)	72 (71.3)	
**Metastasis at diagnosis**, ***n* (%)**			0.05
Yes	79 (75.9)	64 (63.4)	
No	25 (24.1)	37 (36.6)	
**Metachronous metastasis**, ***n* (%)**			0.06
Yes	25 (24.1)	36 (35.6)	
No	79 (75.9)	65 (64.4)	
**Liver metastasis at diagnosis**, ***n* (%)**			0.002
Yes	9 (8.7)	0 (0)	
No	95 (91.3)	101 (100)	
**DNA repair mutation**, ***n* (%)**			0.436
Positive	10 (9.6)	5 (5.0)	
Negative	38 (36.5)	38 (37.6)	
Unknown	56 (53.9)	58 (57.4)	
**Hemoglobin**, ***n* (%)**			0.073
<12 g/dL	30 (28.8)	20 (19.8)	
≥12 g/dL	61 (58.7)	74 (73.3)	
Unknown	13 (12.5)	7 (6.9)	
**PSA**, ***n* (%)**			0.094
<17 ng/mL	46 (44.2)	56 (55.4)	
≥17 ng/mL	58 (55.8)	45 (44.6)	
**First-line treatment**, ***n* (%)**			0.080
ARPI	43 (41.3)	45 (44.6)	
Only ADT	34 (32.7)	42 (41.6)	
Docetaxel	27 (26.0)	14 (13.8)	

Statistical significance was considered at *p* < 0.05. Note: ECOG, Eastern Cooperative Oncology Group Performance Status; PSA, prostate-specific antigen; ADT, androgen deprivation therapy; ARPI, androgen receptor pathway inhibitor.

**Table 2:** Univariate analysis of prognostic factors for radiographic progression-free survival (rPFS).

Subgroup	Median rPFS (Months)	95% CI	*p*-Value
**All Patients**	23.8	18.6–29.0	NA
**Bone pain at presentation**			<0.001
Present	16.9	13.3–20.4	
Absent	29.5	25.2–33.8	
**High** **-** **volume disease**			<0.001
With pain presentation	16.7	13.4–20.0	
Without pain presentation	29.0	20.3–37.7	
**Low** **-** **volume disease**			<0.001
With pain presentation	21.0	13.8–28.1	
Without pain presentation	29.5	24.2–34.8	
**Age**			0.623
<70 years	28.2	23.5–32.9	
≥70 years	21.7	18.2–25.3	
**DNA repair mutation**			0.064
Positive	13.8	9.2–18.4	
Negative	21.7	14.1–29.3	
Unknown	27.5	23.1–31.9	
**Metastasis at diagnosis**			0.635
Yes	23.2	17.7–28.7	
No	25.1	15.4–34.8	
**Disease volume**			0.050
High	19.5	15.9–23.1	
Low	27.1	21.7–32.6	
**Metachronous metastasis**			0.654
Yes	25.1	15.4–34.9	
No	23.2	17.7–28.7	
**Liver metastasis at diagnosis**			0.011
Yes	14.9	5.9–29.0	
No	25.1	20.0–30.2	
**Lymph node metastasis at diagnosis**			0.792
Yes	24.2	19.7–28.6	
No	19.6	9.1–30.0	
**Lung metastasis at diagnosis**			0.967
Yes	26.2	20.4–32.1	
No	23.6	17.7–29.5	
**Hb**			0.002
<12 g/dL	16.9	14.5–19.3	
≥12 g/dL	27.5	23.9–31.1	
**LDH**			0.141
<1.5× ULN	26.2	20.2–32.3	
≥1.5× ULN	20.3	13.9–26.8	
**ECOG PS**			0.094
0	29.0	26.7–31.3	
≥1	20.3	17.8–22.9	
**Albumin**			0.002
≥3.5 g/dL	27.1	23.4–30.8	
<3.5 g/dL	14.7	8.6–20.8	
**ALP**			0.040
<ULN	27.1	17.9–36.3	
≥ULN	19.5	17.5–21.5	

Statistical significance was considered at *p* < 0.05. Note: CI, confidence interval; NA, not applicable; Hb, hemoglobin; LDH, lactate dehydrogenase; ULN, upper limit of normal; ECOG, Eastern Cooperative Oncology Group Performance Status; ALP, alkaline phosphatase.

**Table 3:** Multivariate analysis of prognostic factors for radiographic progression-free survival (rPFS).

Variable	HR	95% CI	*p*-Value
Presentation with pain	2.37	1.29–4.34	0.005
High-volume disease	0.67	0.36–1.27	0.199
Liver metastasis	3.07	1.16–8.15	0.024
Hb < 12 g/dL	2.00	1.90–3.10	0.005
Albumin < 3.5 g/dL	1.99	1.06–3.76	0.034
ALP ≥ ULN	1.25	0.71–2.20	0.447

Statistical significance was considered at *p* < 0.05. Note: HR, hazard ratio; CI, confidence interval; Hb, hemoglobin; ALP, alkaline phosphatase; ULN, upper limit of normal.

## Data Availability

The data presented in this study are available from the corresponding author upon reasonable request. The data are not publicly available because they contain information that could compromise patient privacy.
